# Inhibition of PI3K/AKT molecular pathway mediated by membrane estrogen receptor GPER accounts for cryptotanshinone induced antiproliferative effect on breast cancer SKBR-3 cells

**DOI:** 10.1186/s40360-020-00410-9

**Published:** 2020-05-01

**Authors:** Danning Shi, Piwen Zhao, Lixia Cui, Hongbo Li, Liping Sun, Jianzhao Niu, Meng Chen

**Affiliations:** 1 School of Life Sciences, Beijing University of Chinese Medicine, No. 11, Bei San Huan Dong Lu, Chaoyang District, Beijing, 100029 China; 2 Dongfang Hospital, Beijing University of Chinese Medicine, Beijing, 100078 China; 3 School of Traditional Chinese, Beijing University of Chinese Medicine, Beijing, 100029 China

**Keywords:** Cryptotanshinone, GPER, Breast cancer, Cell cycle, Cyclin, PI3K/AKT, Molecular pathway

## Abstract

**Background:**

Breast cancer is the most frequently diagnosed malignancy among women and the second leading cause of cancer death worldwide. Among which nuclear estrogen receptor (nER) negative breast cancer is always with much poor prognosis. Recently, membrane G protein coupled estrogen receptor (GPER), a newly recognized estrogen receptor has been documented to take essential part in the development and treatment of breast cancer. The present study was designed to investigate the anti nER negative breast cancer effect of cryptotanshinone (CPT), an important active compound of traditional Chinese medicine Danshen and its possible molecular pathway.

**Methods:**

The following in vitro tests were performed in nER negative but GPER positive breast cancer SKBR-3 cells. The effect of CPT on cell proliferation rate and cell cycle distribution was evaluated by MTT cell viability test and flow cytometry assay respectively. The role of PI3K/AKT pathway and the mediated function of GPER were tested by western blot and immunofluorescence. Technique of gene silence and the specific GPER agonist G-1 and antagonist G-15 were employed in the experiments to further verify the function of GPER in mediating the anticancer role of CPT.

**Results:**

The results showed that proliferation of SKBR-3 cells could be blocked by CPT in a time and dose dependent manner. CPT could also exert antiproliferative activities by arresting cell cycle progression in G1 phase and down regulating the expression level of cyclin A, cyclin B, cyclin D and cyclin-dependent kinase 2 (CDK2). The antiproliferative effect of CPT was further enhanced by G-1 and attenuated by G-15. Results of western blot and immunofluorescence showed that expression of PI3K and p-AKT could be downregulated by CPT and such effects were mediated by GPER which were further demonstrated by gene silence test.

**Conclusion:**

The current study showed that the antiproliferative action of CPT on SKBR-3 cells was realized by inhibition of GPER mediated PI3K/AKT pathway. These findings provide further validation of GPER serving as useful therapeutic target.

## Background

Breast cancer becomes one of the most common malignancies and an important public health problem worldwide. It also stands in the second place among the most common causes of death from cancer in women nowadays [1]. The following approaches including surgery, radiation-therapy and chemo-therapy have contributed much to increase the survival rate of patients with breast cancer [2]. But nevertheless, the prognosis of some kinds of breast cancer, especially nuclear estrogen receptor (nER) negative breast cancer remains poor [3]. Thus, further exploration on the effective therapeutic methods against breast cancer are still required.

Besides the above clinical approaches in breast cancer treating, traditional Chinese medicine as well as its active components could also play prominent roles so far. As major bioactive compounds in *Salvia miltiorrhiza Bunge* (Danshen), tanshinones, especially tanshinone I and Tanshinone IIA have been proved to exert inhibitory action on proliferation and migration of breast cancer cells effectively [4–7]. Recently, cryptotanshinone (CPT), another important kind of active component in Danshen began to attract much attention due to its anti-inflammatory [8], anti-bacterium [9] and antitumor effects [10–12]. Among which the antitumor function has been paid much concern and it has also been documented that CPT could inhibit proliferation and promotes apoptosis of breast cancer cells [12]. However, the pharmacological mechanism, especially the molecular pathway of its effect still remains unclear and requires further study. The estrogen-like activity of CPT is also expected comparing its structure with estradiol (see Additional file 1). It was reported that phytoestrogens were plant-derived di- or poly-phenolic compounds which possess estrogenic or antiestrogenic activities due to their structural similarity with 17β-estradiol [13]. An estrogen receptor elements (ERE)-dependent luciferase reporter assay reported by Oche, B et, al [14] has already indicated that CPT could perform phytoestrogenic activity via estrogen receptor (ER) α and ERβ.

Estradiol is a key hormone in the development of breast cancer [15]. Estrogen receptor (ER) plays vital roles in mediating the action of estrogen on proliferation of cells in different target tissues under both physiological and pathological conditions [16]. A key function of estrogen receptor has been reported in the proliferation and migration of breast cancer cells [17]. Besides the classical nuclear estrogen receptor α and β (ERα and ERβ), recently a new kind of membrane estrogen receptor known as G protein-coupled estrogen receptors (GPER) attracted much attention and has been recognized as a major mediator of the rapid cellular effects induced by estrogen throughout the body [18, 19]. For approximately 30% of primary breast cancers are nER negative, GPER is now considered to be a possible target point in cancer therapy, especially in those nER negative breast cancer cells. Increasing evidence revealed that GPER and its mediated signal pathway are involved in the proliferation of breast cancer cells [20, 21].

Crucially, the proliferation of cancer cells depends on the cell cycle. Cell cycle regulation is the major regulatory mechanism of cell growth which is modulated by several types of cyclin and cyclin-dependent kinase (CDK). Cell cycle arrest has been found in a number of cell lines after chemotherapy and its dysregulation is a trait of tumor cells [22]. Antitumor agents may cause a cell cycle arrest in various phases by regulating the cell cycle machinery [23]. As regards to the molecular mechanism of proliferation and cell cycle regulation of human cells, the phosphatidylinositide 3-kinase (PI3K)/AKT signaling pathway has been reported to be an essential route. And in some estrogen related cancers, the PI3K/AKT pathway is mediated by membrane GPER [24]. Akt is a downstream mediator which could be activated by PI3K signaling and then initiates a series of biological effects on proliferation and apoptosis of breast cancer cells. Since activation of PI3K/AKT signal transduction is a main force that drives cell growth, down-regulation or blockage of PI3K and AKT function may be crucial for cancer therapy [25].

In summary, CPT might be a potential GPER targeting medicine for the treatment of nER-negative breast cancer. The current in vitro study was carried out to test the anti nER negative breast cancer effect of CPT and its potential molecular mechanism via GPER mediated PI3K/AKT pathway. GPER as a potential target in nER-negative breast cancer treatment will be expected.

## Methods

### Reagents

Cryptotanshinone (CPT, purity>98%, molecular weight 296.36) was obtained from National Institutes for Food and Drug Control (Beijing, China). 2.964 mg CPT was dissolved in 1 ml dimethyl sulfoxide (DMSO, Sigma, USA) to make a 10 mM stock solution which was then added to the medium at the indicated concentrations. The methyl thiazolyl tetrazolium (MTT) was obtained from Keygen (Nanjing, China). RPMI-1640 medium and 0.05% trypsin were from Gibco (USA) and Fetal bovine serum was from Corning Cell Gro (Australia). Lipofectamine® 2000 was from Invitrogen (Grand Island, NY, USA). GPER specific agonist G-1 and antagonist G-15 were from Cayman Chemical (Michigan, USA). The following antibodies were used: GPER, PI3K(p85), cyclin A, cyclin B, cyclin D, CDK2 (Abcam, USA), phospho-Akt (p-AKT, Ser473, Cell signaling, Boston, MA, USA). GPER siRNA, non-target siRNA and PI3K inhibitor LY294002 were obtained from Santa Cruz Biotechnology (Texas USA). Fluorescein-Conjugated Goat anti-Rabbit IgG(H + L) was from ZSGB-BIO (Beijing, China) and DAPI was from Solarbio (Beijing, China).

### Cell culture

The human breast cancer nER negative SKBR-3 cells obtained from the National Infrastructure of Cell Line Resource (Beijing, China) were maintained in RPMI-1640 medium supplemented with 10% fetal bovine serum, 100 μg/ml streptomycin and 100 U/ml penicillin at 37 °C in a 5% CO_2_ atmosphere.

### MTT cell viability assay

MTT (3-[4,5-dimethylthiazol-2-yl]-2,5-diphenyltetrazolium bromide) cell viability assay was performed as already described [26]. Briefly, cells were seeded in 96-well plates at a density of 5 × 10^3^ cells per well in the growth medium. After 12 h, the cells were treated with 1–10 μM CPT or 0.2% DMSO as a control. 24 h or 48 h later, 15 μL MTT reagent (1 mg/mL) was added into each well and the plates were incubated for an additional 4 h in the dark. Subsequently, the supernatant was removed and the cells were lysed in 150 μl DMSO. The optical density was at a measuring wavelength of 490 nm and a reference wavelength of 570 nm using a plate reader (Multiskan GO, ThermoFisher Scientific, USA).

### Analysis of cell cycle distribution

After treatment with 5–10 μM CPT for 48 h, SKBR-3 cells were removed using 0.05% trypsin. Samples were then washed with phosphate-buffered saline (PBS) for 10 min and stained with 50 μg/ml PI (Sigma, USA) and 250 μg/ml RNase in PBS for 30 min at room temperature in the dark. Subsequently, fluorescence activated cell sorting analysis was performed by FCM (BD FACSCanto II, Becton, Dickinson and Company, NJ, USA).

### Small interfering RNA (siRNA) transfection

SKBR-3 cells were seeded in 6-well cell culture plates. At 80–90% confluent, the cells were transfected with 33 nM GPER siRNA or labeled non-target siRNA as control using Lipofectamine® 2000 transfection reagent according to the manufacture’s instruction. After 24 h, the medium was replaced with fresh medium and the cells were cultured for an additional 24 h before Western blot analysis of GPER expression and MTT cell viability assay.

### Western blot analysis

Exponentially growing cells were seeded in a 6-well plate. 24 h after seeding, the SKBR-3 cells in the logarithmic growth phase were incubated in the absence or presence of CPT for 48 h. 1 μM G-1 or G-15 was added together with CPT in G-1 or G-15 treating group. Then the cells were washed twice with ice-cold phosphate-buffered saline (PBS) and placed in RIPA buffer (Applygen, Beijing, China) supplemented with 1% protein phosphatase inhibitor (All-in-one,100×, Solarbio, Beijing, China) immediately prior to use. Total protein extracts were severally collected in different centrifuge tubes and centrifuged at 12000 rpm for 10 min at 4 °C. Supernatant was recovered subsequently. Then the protein concentration was quantified using bicinchoninic acid (BCA) protein assay kit (Solarbio, Beijing, China). Equal quantities of protein (50 μg) from each sample were separated by 10% sodium dodecyl sulfate-polyacrylamide gel electrophoresis (SDS-PAGE) and then transferred to polyvinylidene fluoride (PVDF) membranes. The membranes were blocked with 5% skim milk dissolved in tris-buffered saline with Tween-20 (TBS-T) (1X TBS, 0.1%Tween 20) for 2 h at room temperature. Western blot analyses were performed using the following primary antibody dilutions: Rabbit anti human cyclin A (1:1000), cyclin B (1:10,000), cyclin D (1:10,000), CDK2 (1:10,000), PI3K (1:1000), GPER (1:250) and mouse anti β-actin (1,10,000). All the above antibodies were diluted in 5% skim milk, respectively. In addition, rabbit anti human p-AKT was diluted for 1:2000 in 5% BSA according to introduction. The membranes were incubated with the above primary antibody dilutions overnight at 4 °C and then washed with TBST for 30 min and incubated with the secondary antibody, horseradish peroxidase-labeled goat anti-rabbit IgG or goat anti-mouse IgG (15,000, Proteintech Group, Inc., China) at 37 °C for 1 h. After additional washes, the membranes were incubated using an enhanced chemiluminescence kit (ECL, Applygen, Beijing, China) and scanned in the multifunctional molecular imaging system (Azure C-Series C600, USA). At last, the bands were collected and analyzed by Image J software (version 1.48, National Institutes of Health, USA).

### Immunofluorescence (IF) assay

Cells were seeded in 8 well chamber slide system (Thermos Fisher, NY, USA) at a density of 1 × 10^4^ per well. Then the cells in the logarithmic growth phase were treated with indicated concentration of CPT with or without G-1 or G-15. Following incubation for another 24 h, the cells were fixed with 4% paraformaldehyde for 30 min, permeabilized with 0.02% Triton-X-100 for 20 min and blocked with 5% goat serum for 30 min. Then the cells were incubated with specific primary antibodies at 4 °C overnight followed by fluorescein isothiocyanate (FITC) labeled secondary antibody for 1 h away from light at room temperature. After washed with PBS, DAPI was used for nucleus staining. Images were visualized using an inverted fluorescence microscope (IX71, Olympus, Japan) and the mean fluorescence intensity was analyzed by Image J software.

### Statistical analysis

Data are expressed as mean ± S.D. of three independent tests at least for each experiment. To compare means among multiple groups, one-way ANOVA followed by the LSD-t multiple comparison test was performed in this study. The statistical analyses were performed using SPSS 20.0 (SPSS, Inc., Chicago, IL, USA) for Windows. The significant difference was confirmed with *P* < 0.05.

## Results

### CPT suppressed SKBR-3 cell growth in a dose-time dependent manner

**Fig. 1:**
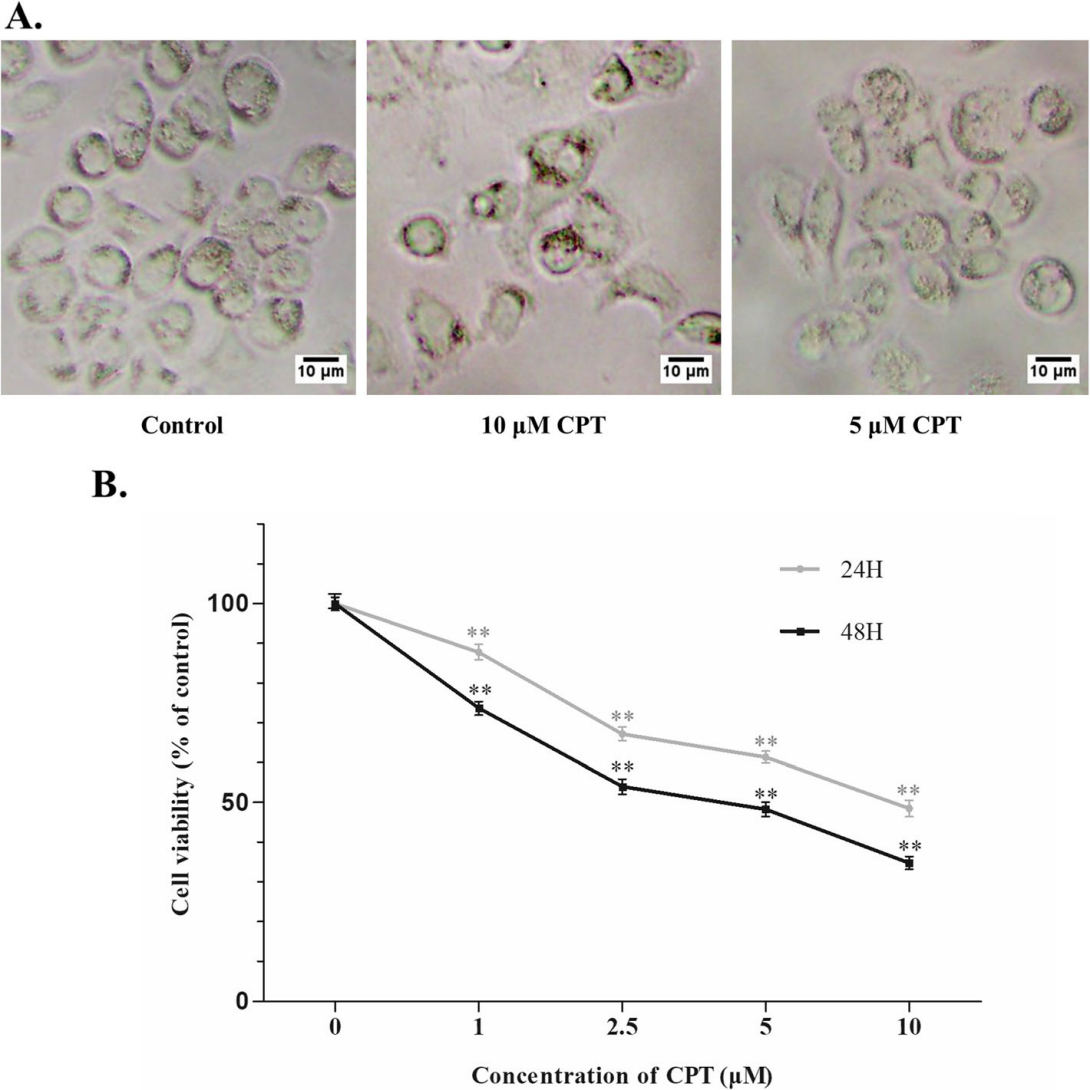
Inhibitory effect of CPT on SKBR-3 cell viability. **a** SKBR-3 cell morphology under inverted microscope (magnification, × 200) after CPT treating for 24 h with 0.2% DMSO as control. Scale bar represents 10 μm. **b** SKBR-3 cells were treated with indicated concentrations of CPT for 24 h or 48 h and cell viability was tested using MTT viability assay. The results are means of three independent replicates ± S.D. ^**^P values < 0.01 vs control group were considered as statistically significant

The viability of SKBR-3 cells decreased significantly after CPT treating for 24 h (Fig. 1a). By using MTT cell viability assay, we demonstrated that CPT could significantly decrease the viability of SKBR-3 cells in a dose and time dependent manner (Fig. 1b). Treatment with 1 μM, 2.5 μM, 5 μM and 10 μM CPT for 24 h and 48 h resulted in 87.8 and 73.7%, 67.2 and 54.0%, 61.4 and 48.3%, 48.5 and 34.8% cell viability compared with control group respectively.

### CPT inhibited SKBR-3 cells viability through a GPER-mediated manner

**Fig. 2:**
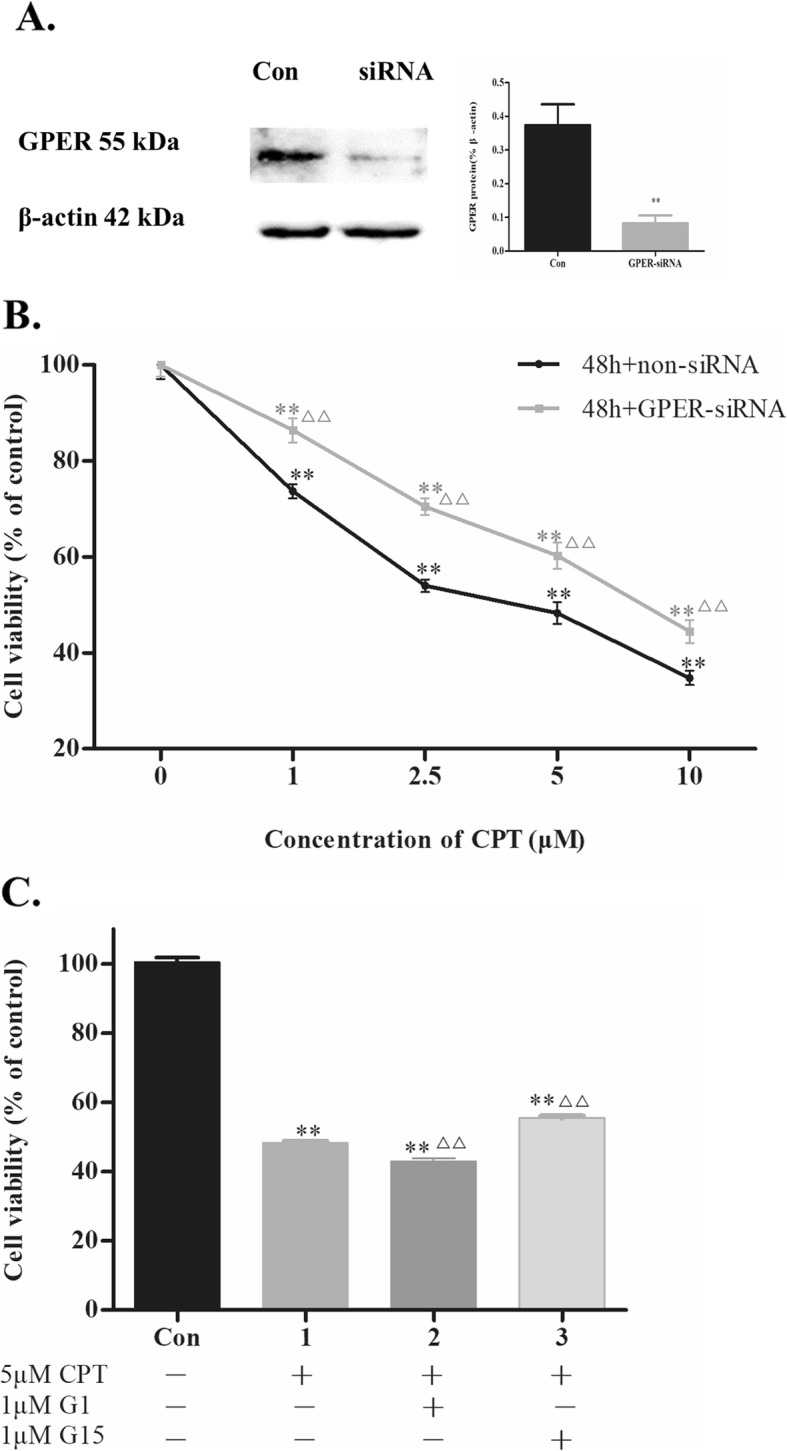
CPT inhibits SKBR-3 cell viability via GPER mediated pathway. **a** GPER expression in GPER siRNA transfected SKBR-3 cells were tested by Western blot with non-siRNA transfection as control. ^**^P < 0.01 vs control group. **b** Viability of GPER knocked down SKBR-3 cells was tested by MTT viability assay. The results are means of three independent replicates ± S.D. ^**^P < 0.01 vs control group or ^*△△*^*P* < 0.01 vs non-siRNA group were considered as statistically significant. **c** Viability of SKBR-3 cells treated by CPT with G1 or G15 was tested by MTT viability assay. The results are means of three independent replicates ± S.D. ^**^P < 0.01 vs control group or ^*△△*^*P* < 0.01 vs 5 μM CPT treated group were considered as statistically significant

To further demonstrate the function of GPER in CPT induced inhibitory effect on cells viability, we knocked down GPER expression by GPER siRNA transfection. The result showed a decreased expression of GPER in SKBR-3 cells after transfected with siRNA targeting GPER (Fig. 2a). Then we tested cell viability affected by CPT in GPER knocked down cells. The results indicated that knockdown of GPER abolished the decrease of cell viability induced by CPT treating for 48 h (Fig. 2b). In addition, viability of SKBR-3 cells was significantly lower when treated by 5 μM CPT together with G-1 while much higher when treated together with G-15 (Fig. 2c).

### CPT induced cell cycle arrest in SKBR-3 cells

**Fig. 3:**
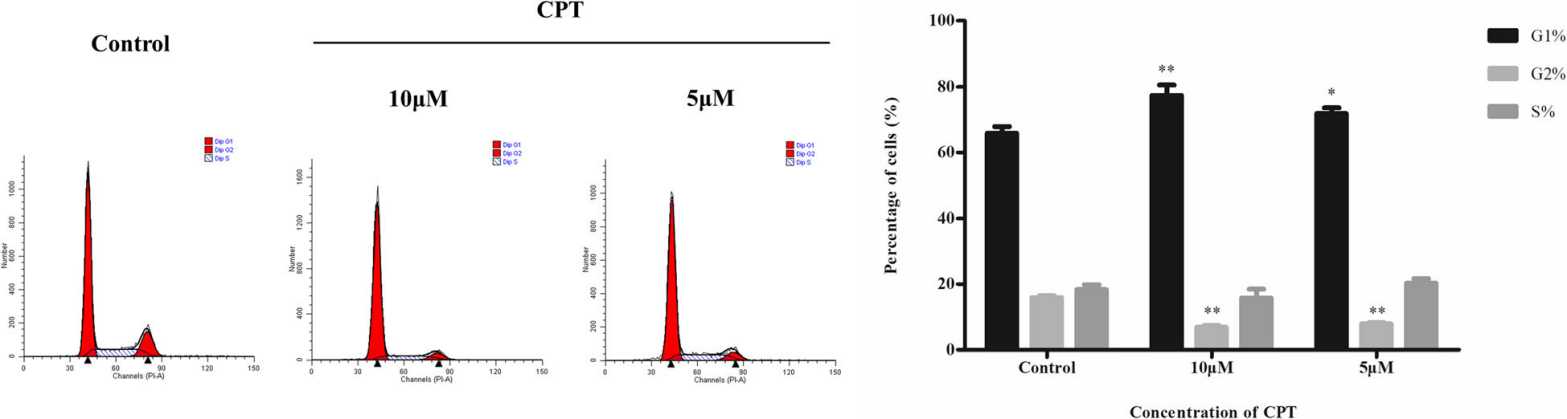
Effect of CPT treating for 48 h on cell cycle distribution in SKBR-3 cells. The results are means of three independent replicates ± S.D. ^*^P < 0.05 or ^**^P < 0.01 vs control group were considered as statistically significant

Cell cycle is the basis of cell proliferation. Inducing cell cycle arrest could be a crucial way to inhibit the development of tumor cells. As shown in Fig. 3, an increase of the percentage of SKBR-3 cells in G1-phase to 71.86 ± 3.03% and 77.35 ± 5.56% was observed with 5 μM and 10 μM CPT treating respectively compared with the control group, 65.83 ± 3.47%. In the meanwhile, percentage of SKBR-3 cells in G2-phase was decreased from 15.86 ± 1.02% in control group to 7.87 ± 0.75% and 6.88 ± 0.87% in 5 μM and 10 μM CPT treating groups respectively. The results revealed that CPT induced suppression of breast cancer SKBR-3 cell growth might be associated with a GPER mediated G1-phase arrest.

### CPT regulated the expression levels of cell cycle-associated proteins in SKBR-3 cells

**Fig. 4:**
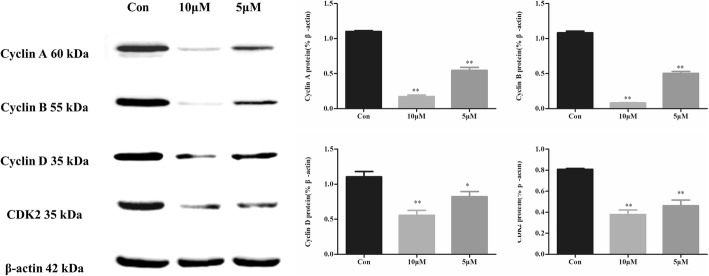
The expression of cell cycle-associated proteins in SKBR-3 following treatment with CPT for 48 h. The results are means of three independent replicates ±S.D. ^*^P < 0.05 or ^**^P < 0.01 vs control group were considered as statistically significant

Following CPT treating for 48 h, the expression levels of cyclin A, cyclin B, cyclin D and CDK2 decreased in a dose-dependent manner (Fig. 4). Especially, cyclin B expression which is closely related to the G2/M-phase transformation of the cell cycle, presented the most obvious decreasing after CPT treatment.

### CPT downregulated PI3K/AKT signaling pathway in SKBR-3 cells

**Fig. 5:**
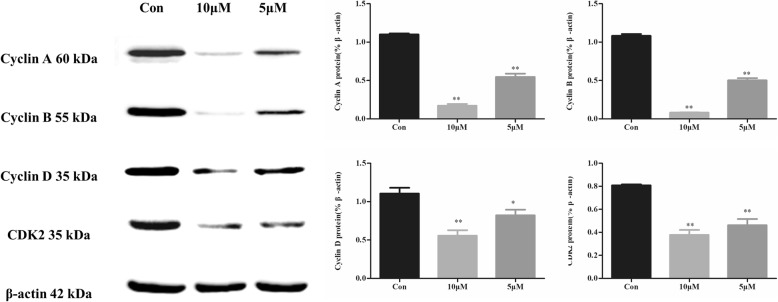
The expression of PI3K and p-AKT in SKBR-3 cells treated by CPT for 48 h. The results are means of three independent replicates ± S.D. ^*^P < 0.05 vs control group were considered as statistically significant

In order to clarify whether GPER mediated PI3K/AKT signaling pathway was involved in CPT induced cell viability inhibition and cell cycle arrest in SKBR-3 cells, we tested the expression of PI3K and p-AKT after CPT treating. Image analysis demonstrated an obvious dose-dependent reduction of p-AKT and PI3K expression in response to CPT treating (Fig. 5).

### CPT induced changement of PI3K, p-AKT and cell cycle-associated protein expression in SKBR-3 cells is realized in a GPER mediated manner

**Fig. 6:**
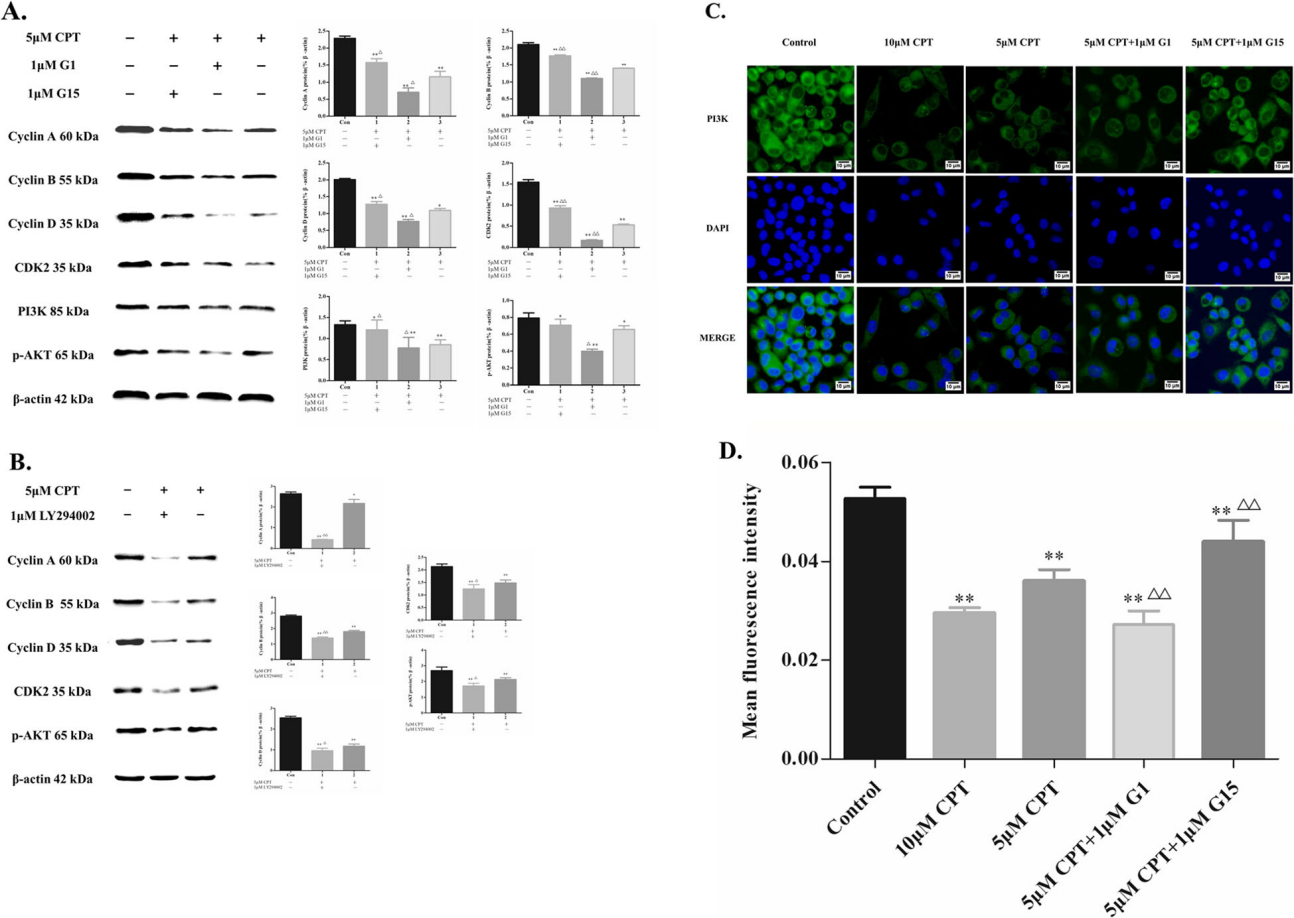
Regulation of PI3K/AKT signaling pathway mediated by CPT via GPER in SKBR-3 cells. **a** The expression of cyclin A, cyclin B, cyclin D, CDK2, PI3K and p-AKT following G-1, G-15 treating together with 5 μM CPT. **b** The expression of cyclin A, cyclin B, cyclin D, CDK2 and p-AKT following LY294002 treating together with 5 μM CPT. **c** Effect of CPT treatment for 24 h on PI3K expression by using immunofluorescence assay. Nuclei were counterstained with DAPI (blue). (magnification, × 200, Scale bar represents 10 μm). **d** The mean fluorescence intensity of PI3K by IF assay was analyzed using Image J software. The results are means of three independent replicates ± S.D. ^**^P < 0.01 or ^*^P < 0.05 vs control group, ^*△△*^*P* < 0.01 vs 5 μM CPT treating group were considered as statistically significant

As shown in Fig. 6a, treatment of SKBR-3 cells with G-1 or G-15 together with 5 μM CPT resulted in a decreasing and increasing expression level of PI3K and p-AKT respectively. Application of LY294002 resulted in a reduction of p-AKT and cell cycle associated proteins expression (Fig. 6b). These findings supported the hypothesis that inhibition of SKBR-3 by CPT was realized by the GPER mediated suppression of PI3K/AKT signaling transduction.

Expression of PI3K was also shown using IF assay in Fig. 6c. After analyzing by image J software, the mean fluorescence intensity was shown in Fig. 6d. The results confirmed that expression of PI3K could be inhibited by CPT in a dose dependent manner. When G-1 was used together with CPT, PI3K expression further decreased. On the contrary, PI3K expression increased significantly after using G-15.

## Discussion

Breast cancer is the most common cancer displaying a high mortality rate among women. And there was an estimated 5-year survival rate as 80% in developed countries to below 40% for developing countries [27]. Clinical and laboratory evidences have indicated that nER-negative breast cancer was resistant to several anticancer drugs [28, 29]. In view of its poor prognosis, clinical therapy towards nER-negative breast cancer was still a big challenge. Therefore, there is an urgent need to find much effective treating methods towards nER-negative breast cancer.

It was reported that many kinds of breast cancer cells, including some nER negative breast cancer cells such as SKBR-3 cells were membrane GPER positive [30]. In vitro researches on the effect of GPER in breast cancer treating claimed that GPER might function as a tumor suppressor in breast cancer cells [21, 31–33]. GPER was a seven-transmembrane protein which has been proved to play mediating roles in rapid non-genomic signal transduction responding to estrogen [19]. The discovery of GPER as a receptor for estrogens brought a new breakthrough point to explain the mechanism of the effects of estrogen or estrogenic substances on growth and migratory process of target cells, especially in the absence of classic nER.

Regarding the anticancer agent, traditional Chinese medicine has attracted much attention worldwide in recent years. There has also been an increasing interest in the anticancer activity of tanshinones including cryptotanshione, a cell permeable diterpene quinone, which is one of the major bioactive compounds of Danshen [12]. Based on its structural similarity to 17β-estradiol, the phytoestrogenic activity of CPT was expected. The current research focused on its antiestrogenic effects via GPER mediated molecular pathway in nER-negative SKBR-3 cells.

The results of our study showed that CPT were capable of inducing proliferation inhibition and cell cycle arrest in SKBR-3 cells via GPER in a time and dose dependent manner. Concretely, the cell viability assay indicated that CPT could inhibit proliferation of SKBR-3 cells. Silencing GPER with specific siRNA abolished CPT induced decrease of cell viability. The results clearly indicated that activation of GPER was an important mechanism contributing to the anticancer activity of CPT in SKBR-3 cells. Application of GPER selective agonist G-1 and antagonist G-15 gave us more information about the function of GPER mediating the effect of CPT on cell proliferation. G-1 was reported to suppress proliferation of ovarian and breast cancer cells in vitro [34] and G-15 was reported to inhibit GPER-mediated function on cell proliferation in vitro [35]. In accordance with these previous studies, the inhibitory effects on cell proliferation induced by CPT was further increased with the application of G-1 and decreased with G-15.

A large section of researches have indicated that cell cycle arrest was closely linked to cell proliferation inhibition in cancer cells [25, 36, 37]. It was of much significance to deregulate the cell cycle in cancer treatment. Therefore, elucidating the inhibitory activity on cell cycle by CPT could help us to understand the mechanism of its anticancer effect. The results of our study claimed that the protein expression of cyclin A, cyclin B, cyclin D and CDK2 were dose-dependently decreased after CPT treating. It was a recognized concept that cells initiate their fate decisions in G1-phase. In early G1-phase, cyclin D is a main factor which could drive the cell cycle to S-phase and the DNA replication could be induced [38]. Hence, CPT induced decrease of cyclin D in SKBR-3 cells might cause a G1-phase arrest which was further demonstrated by FCM test. The expression of cyclin A was mainly related to S-phase and cyclin B to G2/M-phase. Our results indicated that the expression of both cyclin A and cyclin B were inhibited by CPT in a dose-dependent manner. It might also be induced by the G1-phase arrest in cell cycle since large amounts of cells stopped at G1-phase and the cell cycle could not proceed as original.

As a key regulator of cell proliferation and survival, the PI3K/AKT pathway is critical for cell cycle progression. AKT, the Ser/Thr kinase, was the core component of this cascade of events and activation of PI3K results in the phosphorylation of AKT. That is to say, PI3K-produced phospholipids favor the membrane recruitment of AKT which is further self-activated by either 3-phospho-inositidedependent protein kinase 1 (PDK1) or the mechanistic target of rapamycin (mTOR) complex 2 (mTORC2) [7, 39] it was reported that p-AKT signaling was much more activated in breast cancer cells and targeting PI3K/AKT signaling may be considered a prime strategy in cancer treatment [40]. Consistent with these studies, 48 h treatment with CPT showed an inhibitory effect on the expression of both PI3K and p-AKT. PI3K/AKT favors cell proliferation through direct regulation of cell cycle protein, the cyclins [25, 41]. Our current results, the expression of cyclin A, cyclin B, cyclin D and CDK2 further decreased with the specific PI3K inhibitor-LY294002, also significantly indicated that CPT-induced cell cycle arrest was associated with the inhibition of PI3K/AKT activity in SKBR-3 cells. IF assay also showed a CPT induced down regulation of PI3K expression and the results of G-1 and G-15 intervention study further demonstrated that such effect was mediated by GPER.

## Conclusion

In conclusion, the most interesting aspect that could be extrapolated from our results was that CPT could be a promising agent in cancer treating, especially nER negative breast cancer. Such effect of CPT was mainly realized by its GPER mediated regulatory function on PI3K/AKT signaling pathways.

## Supplementary information

**Additional file 1: Figure S1.** The molecular structure of (A) CPT and (B) 17β-estradiol.

## Data Availability

The datasets used and analyzed during the current study are available from the corresponding author on reasonable request.

## Abbreviations

GPER: G protein-coupled estrogen receptor
nER: Nuclear estrogen receptor
ERE: Estrogen receptor element
CDK: Cyclin-dependent kinase
DAPI: Diamidino-2-phenylindole
FCM: Flow cytometer
FITC: Fluorescein isothiocyanate
PI: Propidium iodide
PI3K: Phosphatidyl inositol 3-kinase
AKT: PKB, protein kinase B
