# Supplementary figures and images for: Inhibition of PI3K/AKT molecular pathway mediated by membrane estrogen receptor GPER accounts for cryptotanshinone induced antiproliferative effect on breast cancer SKBR-3 cells
Source: BMC Pharmacol Toxicol. 2020 May 1;21:32. doi: 10.1186/s40360-020-00410-9 (PMC7193699; doi:10.1186/s40360-020-00410-9)

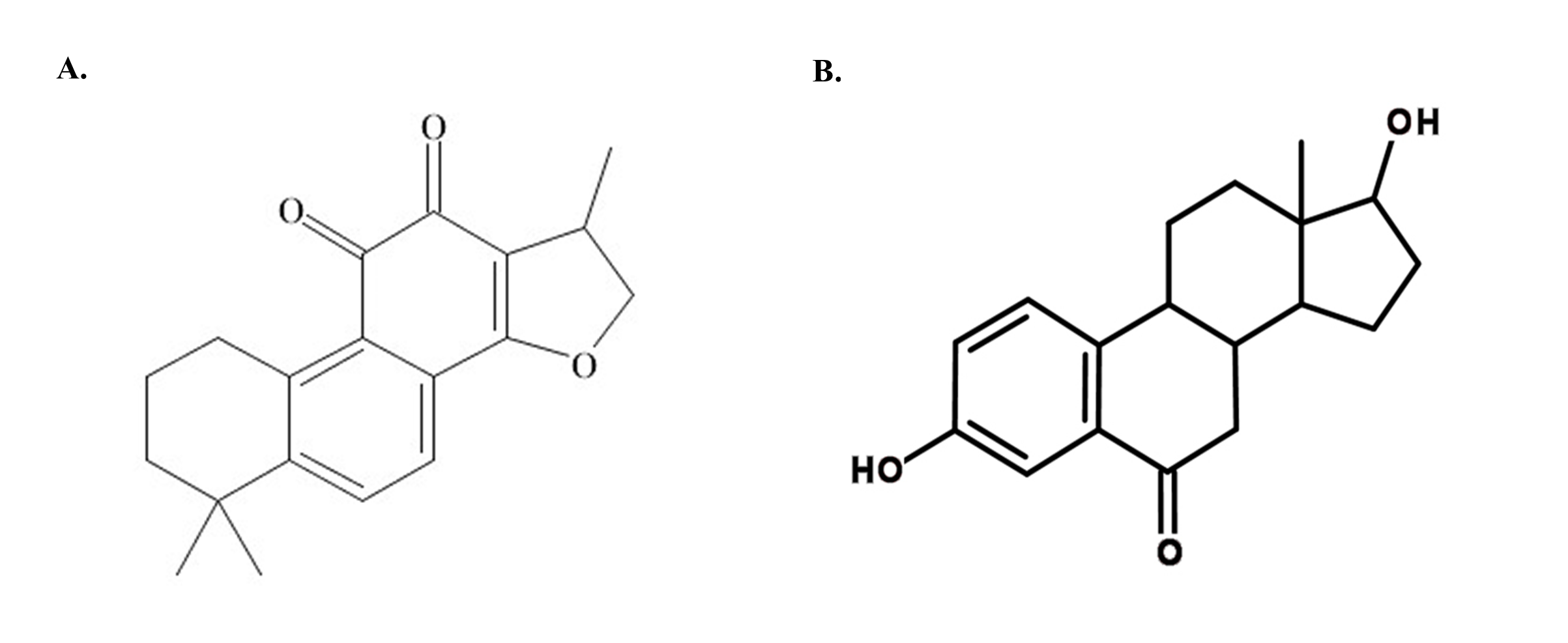

Supplement: Supplementary file 1 — Additional file 1: Figure S1. The molecular structure of (A) CPT and (B) 17β-estradiol. [file 40360_2020_410_MOESM1_ESM.tif]
